# Shifting decision thresholds can undermine the probative value and legal utility of forensic pattern-matching evidence

**DOI:** 10.1073/pnas.2301844120

**Published:** 2023-10-02

**Authors:** William C. Thompson

**Affiliations:** ^a^Department of Criminology, Law & Society, University of California, Irvine, CA 92617

**Keywords:** forensic science, decision threshold, signal detection theory, Bayesian network, error rates

## Abstract

Forensic pattern analysis requires examiners to compare the patterns of items such as fingerprints or tool marks to assess whether they have a common source. This article uses signal detection theory to model examiners’ reported conclusions (e.g., identification, inconclusive, or exclusion), focusing on the connection between the examiner’s decision threshold and the probative value of the forensic evidence. It uses a Bayesian network model to explore how shifts in decision thresholds may affect rates and ratios of true and false convictions in a hypothetical legal system. It demonstrates that small shifts in decision thresholds, which may arise from contextual bias, can dramatically affect the value of forensic pattern-matching evidence and its utility in the legal system.

## Significance

This article demonstrates an important but largely unrecognized truth about forensic pattern analysis: that small shifts in forensic examiners' decision thresholds can dramatically affect their error rates and the probative value of their evidence, which can in turn affect the accuracy of the legal system. For example, small reductions in the threshold for identification, which might plausibly arise from an examiner's exposure to task-irrelevant information, can dramatically increase the risk of convicting an innocent person. This means that the decision process of forensic examiners is not merely a scientific issue; it has important legal ramifications that deserve broader attention.

Forensic pattern analysis requires an examiner to consider two propositions:

*H*—the items being compared have a common source; and
*A*—the items have a different source.

The examiner tries to distinguish these propositions based primarily on evidence, *E*, which consists of two components:

*X* = observations regarding the pattern of the reference sample
*Y* = observations regarding the pattern of the questioned item or trace

Hence, $E=\left(X,Y\right)$.

To evaluate a case, the examiner must consider the probability of *E* under the alternative propositions. In other words, the examiner must make an assessment of two key conditional probabilities: *p(E|H)* and *p(E|A)*.

In some jurisdictions, particularly in Europe, examiners are allowed to report their opinions regarding the ratio of these two conditional probabilities, which is called a likelihood ratio (LR) (1, 2). For example, an examiner might opine that the degree of similarity observed between the reference sample and questioned item is 100, 1000, or even 1 million times more probable if the items have a common source than a different source. Alternatively, or additionally, the examiner might make a verbal statement that is keyed to the LR. If the examiner puts the LR at 1 million or higher, then she might report that the evidence provides “extremely strong support” for the proposition that the items have a common source. A more moderate LR would be characterized with a more modest statement; for example, an LR of 100 is often said to provide “moderate support.”

In the United States, most pattern-matching examiners use categorical scales to report their conclusions. Fingerprint examiners have traditionally reported one of three conclusions: identification, exclusion, or inconclusive. Other disciplines allow additional reporting categories (1, 2). Regardless of the reporting scale, the examiner’s decision to report a categorical conclusion rests on an assessment of the relative value of *p(E|H)* and *p(E|A).* To report “identification,” for example, the examiner must conclude that the ratio *p(E|H)/p(E|A)* is high enough to justify that decision.

In general, this decision is subjective. It occurs entirely in the mind of the examiner: No measurements are recorded, no quantification is attempted, and no objective rules are applied. Examiners are typically trained to follow a procedure known as ACE-V, which stands for analysis, comparison, evaluation, and verification (3), but ACE-V merely specifies certain steps that examiners should take to extract and compare features (such as minutiae in fingerprint impressions), it provides no standards or guidelines for assessing the similarity of features nor for deciding whether they are sufficiently similar to pass a decision threshold.

## Information Examiners Might Consider

The National Commission on Forensic Science (4) drew a helpful distinction between two types of information that an examiner might consider when performing pattern analysis:

- Task-relevant information helps inform evaluation of the two key conditional probabilities*: p(E|H)* and *p(E|A);*
- Task-irrelevant information has no bearing on *p(E|H)* or *p(E|A),* but may affect the examiner’s impression of the priors, *p(H)* and *p(A)*, and thereby influence assessment of *p(H|E)/p(A|E)*.

To illustrate the distinction mathematically, let *CI* designate contextual information about a case. Then,

CI is task-relevant IF: *p(E|H) ≠ p(E|H,CI) OR p(E|A) ≠ p(E|A, CI)*

CI is task-irrelevant IF: *p(E|H) = p(E|H,CI) AND p(E|A) = p(E|A, CI)*

Examples of task-relevant information for pattern-matching examiners include the following:

- That a latent fingerprint was lifted from a curved or irregular surface
- That reference shoe was collected 6 mo after the questioned print was made at a crime scene
- That the writer of a questioned signature may have been intoxicated at the time the document was signed

In each example, the contextual information bears on the probability of making certain observations on the patterns if the items have the same source or if they have a different source. The irregular surface may affect the appearance of the latent print; additional wear during the 6 mo between when the print was made and the shoe was collected may have affected the appearance of the reference print; and the writer’s intoxication may have affected the appearance of the questioned signature. Thus, this information helps the examiner evaluate and assign values to *p(E|H)* and *p(E|A)*.

Examples of task-irrelevant information for a pattern matching examiner include the following:

- The suspect’s criminal history
- That the police believe that the suspect is guilty
- That the suspect confessed
- That the suspect was implicated by other physical evidence at the crime scene
- That another examiner identified the suspect as the source of a print found on a different item at the same crime scene

This type of information may well affect one’s assessment of the probability that *H* or *A* is the correct proposition. Notice, however, that this information does not affect the key conditional probabilities, *p(E|H)* or *p(E|A).* In other words, this information has no bearing on the probability of making specific observations IF the patterns have the same source; or IF they have a different source. The National Commission explained this point using the example of a fingerprint comparison:

If the suspect is the source of the latent print, then a high degree of similarity between the prints is to be expected, regardless of whether the suspect confessed, has an alibi, or has a criminal record. If the suspect is not the source of the latent print, then a low degree of similarity is to be expected, regardless of these other factors.
Because information of this type does not affect the relevant conditional probabilities, it does not help the analyst assess the strength of the inferential connection between the evidence designated for examination and the relevant propositions. It might help the analyst draw conclusions about the propositions, but it does not help the analyst draw conclusions *from the physical evidence that has been designated for examination through correct application of an accepted analytic method*. Any inferences analysts might draw from the task-irrelevant information involve matters beyond their scientific expertise that are more appropriately considered by others in the justice system, such as police, prosecutors, and jurors (4, Appendix).

Although the National Commission urged forensic scientists to avoid considering task-irrelevant information, the temptation to do so may be powerful. Like any human being, forensic scientists surely have a strong desire to make the right decision. Consider a fingerprint examiner who sees a great deal of similarity between two prints but is uncertain whether the similarity is sufficient to make an identification. Learning of other evidence that supports the theory of a common source may bolster the examiner’s confidence that identification is the right decision and consequently increase the chances the examiner will classify the case as an identification.

At least one commentary has suggested that this kind of contextual influence is beneficial for the legal system: “…task-irrelevant contextual information does not necessarily lead to inaccurate decision-making and … subjective interpretations of forensic evidence based upon task-irrelevant contextual information may even promote accurate decision-making” (5). Other commentators have raised concerns, however, that this practice may mislead lawyers and jurors by causing them to “double-count” the evidence (6–8). Jurors may, for example, assume the forensic identification is independent of other evidence against the defendant, not realizing that the forensic identification was made partly because of the other evidence against the defendant. Examiners’ desire to make the right call may thus create a misleading impression of the value of the forensic evidence. This raises an intriguing possibility: Reliance on task-irrelevant information may make a forensic scientist more accurate while simultaneously making the legal system less likely to reach the correct outcome (conviction of the guilty and acquittal of the innocent), a situation that has been called the “criminalist’s paradox” (6–8).

This article explains how and when that might happen. It uses statistical modeling to assess, at a conceptual level, the circumstances under which a forensic examiner’s use of task-irrelevant information will be beneficial or detrimental for the legal system. It attempts to answer the question of whether it is good or bad for forensic scientists to consider task-irrelevant contextual information by linking this important aspect of forensic laboratory practice to a broader analysis of the accuracy of the legal system.

## Modeling Examiners’ Decisions

A forensic examiner’s task can be viewed as a signal detection problem (9–11). The examiner attempts to distinguish same-source and different-source items based on the degree of similarity. While same-source items are generally more similar than different-source items, there is a range of similarity in both categories and their distributions overlap. Examiners set decision thresholds in a manner intended to minimize errors, but errors are inevitable and the ratio between false identifications and false exclusions varies depending on where the decision thresholds are set.

**Fig. 1.**
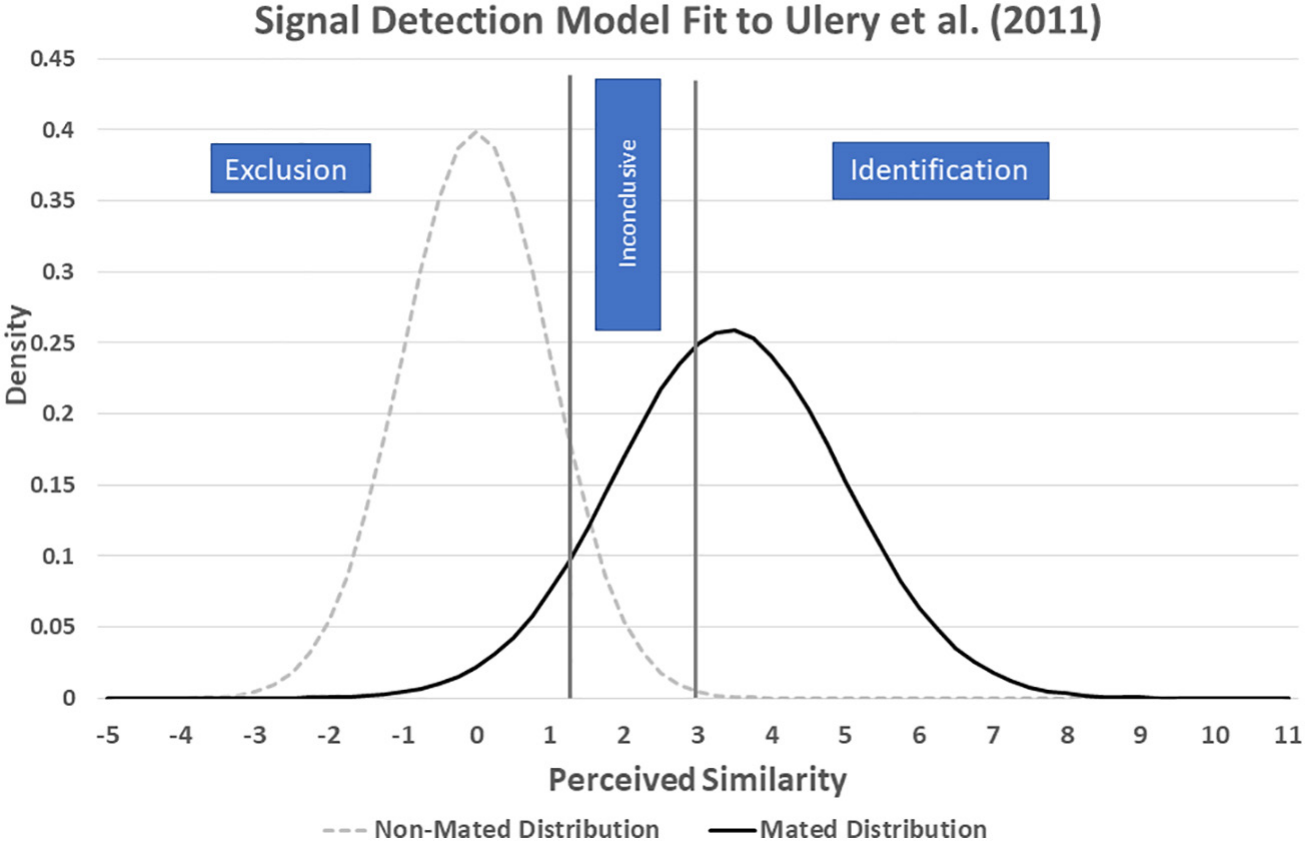
Signal detection model of fingerprint comparison data from Ulery et al. (13). Note: Mated distribution mean 3.52; SD 1.54. Exclusion criterion 1.21; identification criterion 2.97. Numbers on the x-axis represent the distance in standard deviations from the mean of the non-mated distribution.

It is possible to infer parameters of a signal detection model for a forensic discipline by fitting the model to data from black-box studies of examiner accuracy (12). I created the model shown in Fig. 1 by fitting it to data from Ulery et al. (13), the most extensive black-box study of fingerprint examiner accuracy. Following a procedure described by Mannering et al. (12), I began with the assumption that perceived similarity is distributed normally for same-source (mated) and different-source (nonmated) print comparisons and that the variance of the two distributions might vary. To set the scale of the *x* axis, I fixed the different-source (nonmated) distribution with a mean of zero and a SD of one. I used maximum likelihood estimation to infer four additional parameters (the mean and SD of the same-source distribution and the location of two decision criteria). The perceived similarity of the prints being compared must exceed the identification (ID) criterion for the examiner to report an ID and must fall below the exclusion criterion for the examiner to report an exclusion. If the perceived similarity falls between the two decision criteria, the examiner reports the comparison as “inconclusive.” If one of the prints lacks sufficient detail to allow a determination, then the examiner might also deem the comparison inconclusive. For the dataset considered here, however, all of the comparisons involved prints that participants had previously judged to have value for identification (13).

As this model shows, the latent print examiners who participated in the Ulery et al. study (13) set a threshold for identifications that minimized the number of false IDs but resulted in failure to ID a substantial percentage of same-source comparisons. Their exclusion criterion was less stringent, allowing many more false exclusions than false IDs. A substantial percentage of all comparisons was classified as inconclusive.

A key issue for us to consider is whether forensic scientists will set their decision thresholds the same way when performing casework as when participating in a black-box study. Because the decision to identify, or exclude, is made subjectively, without any objective standards, it seems possible, even likely, that the decision criteria will vary depending on the circumstances (14, 15). Research on human judgment indicates that decisions can be influenced by contextual factors without the decision-maker even realizing it (16). Moreover, there is a considerable body of research indicating that exposure to task-irrelevant information can affect forensic examiners’ decisions to “identify” or “exclude” items they compare (17–22). If exposure to task-irrelevant information causes examiners’ decision thresholds to shift, how will that affect the probative value of their evidence?

**Fig. 2.**
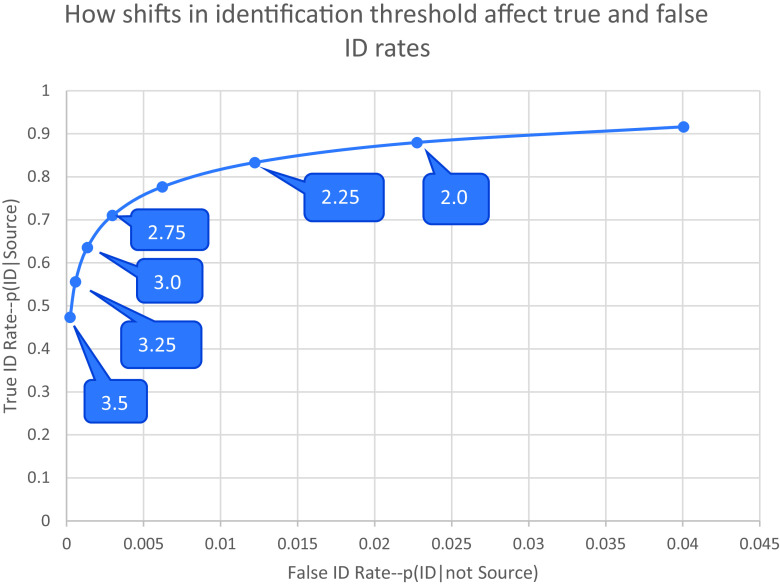
ROC curve showing how the true and false ID rates of a latent print examiner will vary as a function of the identification threshold. The threshold values indicate the distance (in SDs) between the threshold and the mean of the nonmated distribution in the signal detection model shown in Fig. 1.

Fig. 2 is a receiver operating characteristicn (ROC) curve, based on the parameters of the model shown in Fig. 1, that shows how shifts in an examiner’s identification criterion affect the trade-off between true and false identifications. The labels show the position of the identification criterion along the *x* axis of Fig. 1. Decreasing the criterion for an identification causes an examiner to slide upward and to the right along this curve, as the rate of true IDs and false IDs both increase. Raising the criterion for identification causes the examiner to slide downward and to the left on this curve as the rates of true IDs and false IDs both decrease.

**Fig. 3.**
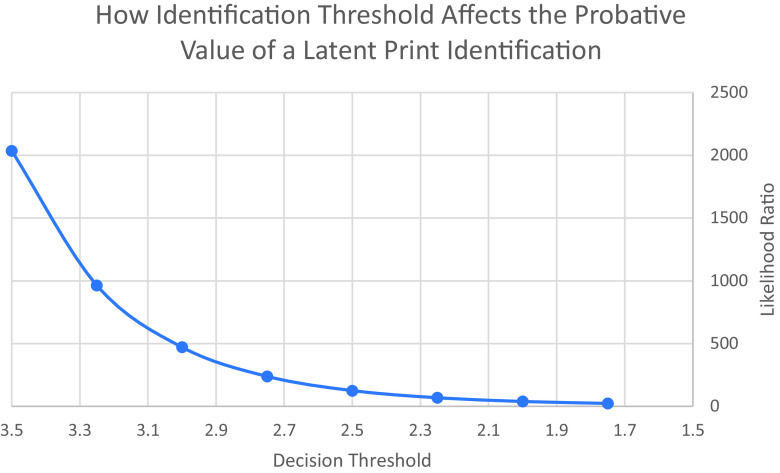
Identification threshold and the LR for an identification.

Fig. 3 shows how this trade-off between true IDs and false IDs affects the probative value of a latent print ID. The traditional measure of the probative value of evidence for distinguishing two propositions is the LR. As a reminder, in this instance, there are two propositions being distinguished: *H*—the items being compared have a common source; and *A*—the items have a different source. The evidence we are evaluating now is a report that the examiner has “identified” the items as having a common source. We can compute the LR that describes the strength of that evidence by simply dividing the true positive rate by the false positive rate, to produce a number showing how much more likely an ID is for same-source comparisons than for different-source comparisons. As Fig. 3 shows, the LR drops rapidly as the examiner decreases the identification threshold. The model shown in Fig. 1 indicated that examiners in the Ulery et al. study (13) set the identification threshold 2.97 SDs above the mean of the nonmated distribution. This threshold produces an LR of about 500. If an examiner were to lower the threshold by about one-quarter SD, to 2.75, however, the LR would be approximately cut in half to around 250. Each additional quarter SD drop in threshold would again cut the LR by about half. If the threshold were dropped to 2 SD, the true ID rate would be 0.88, and the false ID rate would be 0.022, making the LR only 38.7.

The rapid decrease in the LR as examiners lower their decision thresholds appears to be an inevitable consequence of the shape of the distributions of perceived similarity for same source and different source comparisons. So long as perceived similarity is distributed normally, and the decision threshold falls in the area where the distributions overlap, a downward shift will produce a reduction in the LR. The reduction causes an increase in both the true ID rate and the false ID rate, but the true ID rate is generally high, even when the threshold is high, and does not rise very fast. By contrast, the false ID rate starts extremely low but rises much more rapidly (relative to the true ID rate) as the threshold drops. The parameters of the distributions (their means and SDs) affect the overall LRs and how rapidly they change, but the conclusion of this analysis—that downward shifts in threshold reduce the probative value of a forensic ID—does not depend on the parameters; it is a general property of signal detection analysis when the decision criterion falls between overlapping normal distributions.

What this means is that ANY reduction in an examiner’s identification threshold decreases the probative value of a forensic identification. Examiners who lower their thresholds upon learning of other incriminating evidence are reducing the probative value of the evidence that they provide to the legal system when they make an identification. This is true regardless of whether the examiners are aware their thresholds have shifted. And because examiners are largely free to set their thresholds as they wish, it may not be apparent to lawyers and triers-of-fact whether a high or low threshold was set in a given case. This analysis suggests, then, that if we wish to maximize the value of a forensic identification for the legal system, we should encourage examiners to set a high decision threshold and should avoid exposing them to task-irrelevant information that might cause them to lower it.

The question we seek to answer in this article, however, is how shifts in decision threshold affect the accuracy of the legal system. Are there circumstances in which it makes sense to vary decision thresholds to increase the chances that the examiner will make the right call? How might forensic examiners’ decision thresholds affect the balance between true and false convictions? In the next section, this article attempts to answer these questions by building a simple conceptual model of a legal system, using Bayesian networks. While the model greatly simplifies reality, it provides insights regarding the connection between examiners’ decision thresholds and the performance of the legal system and thereby helps us understand the potential legal consequences of examiners’ decision thresholds.

## A Simple Legal System

Bayesian networks (also known as relevance diagrams or influence diagrams) are often used to represent situations in which decisions are made (23). Developed in the 1970s as an alternative to decision trees (24, 25), Bayesian networks provide a graphical and mathematical model of a decision-making task and show how one event might influence another. The relationships among the various events in a Bayesian network can be described mathematically, and the resulting equations can be used to show how changes in the state of one event might affect the probability of other events. Software has been developed to perform the necessary computations, which makes Bayesian network models relatively easy to use for exploring the interconnections between the events that are being modeled.

**Fig. 4.**
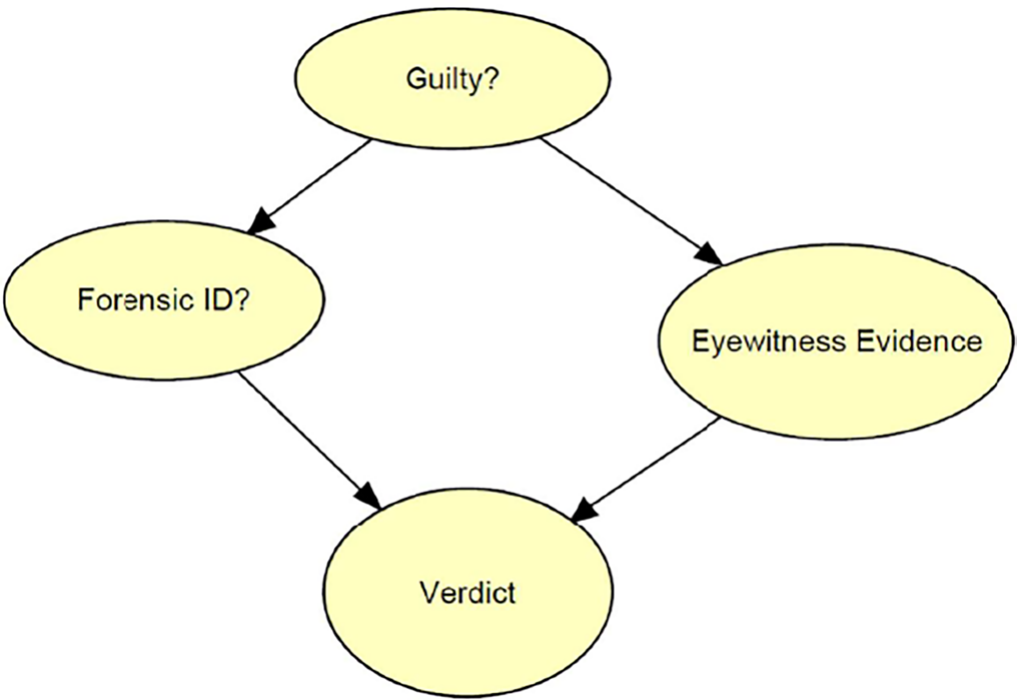
Simple model of a legal system.

Fig. 4 shows the core of a Bayesian network that represents a simplified model of a legal system. In this system, criminal cases are decided based on two types of evidence: eyewitness evidence and forensic pattern-matching evidence. Each suspect processed through this legal system is presented to the eyewitness, who either identifies the suspect as the perpetrator or fails to make an identification. Additionally, a forensic scientist compares trace evidence left by the perpetrator at the crime scene with reference material associated with the suspect (e.g., comparing a latent print with the suspect’s fingerprint) and reports either an identification, exclusion, or inconclusive. The reports of the eyewitness and of the forensic scientist determine the verdict in the case. If both make an identification, then the suspect is convicted; if either fails to make an identification, then the suspect is acquitted; if the eyewitness makes an identification and the forensic evidence is inconclusive, there is a 50% chance of conviction.

The node at the top of the model represents the guilt or innocence of the suspect. The model assumes that some suspects are guilty and some are innocent. The arrows linking the guilt node to the forensic evidence and eyewitness evidence indicate that the guilt or innocence of the suspect influences the state of the forensic and eyewitness evidence. Guilty suspects are more likely to be identified than innocent suspects. The arrows linking the forensic evidence and eyewitness evidence to the verdict show that the two types of evidence jointly determine the verdict.

### Setting Parameters and Assessing System Performance.

**Fig. 5.**
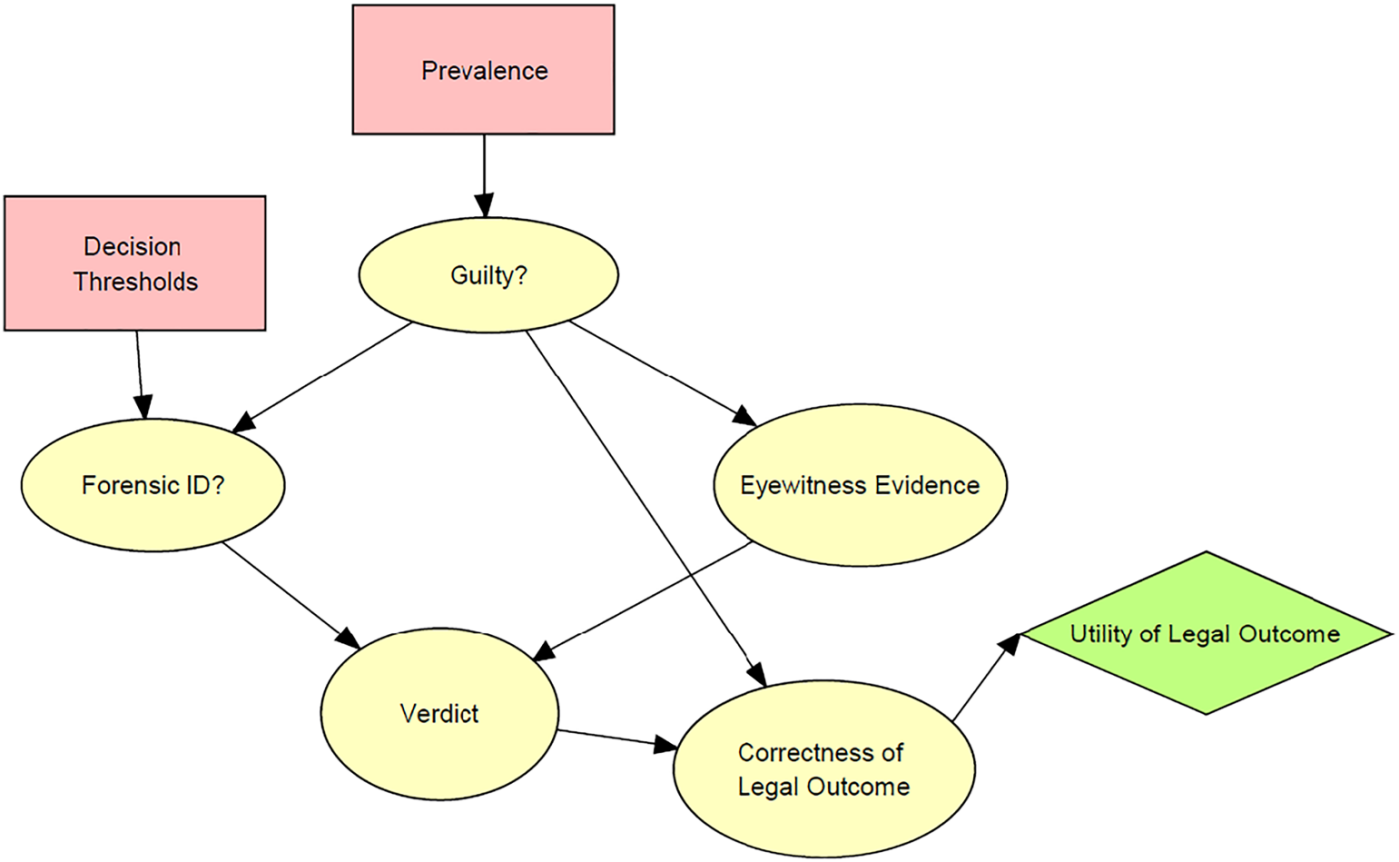
Expanded network with nodes for assessing system performance.

Fig. 5 shows the same basic model with four additional nodes. Two of the nodes are used for assessing system performance. The “Correctness of Legal Outcome” node (to the right of verdict) divides possible legal outcomes into four categories: correct conviction (conviction of a guilty suspect); correct acquittal (acquittal of an innocent suspect); false conviction (conviction of an innocent suspect); and false acquittal (acquittal of a guilty suspect). The assignment of cases to the four categories depends entirely on the state of the parent “Guilty” and “Verdict” nodes. The “Utility of Legal Outcome” node assigns utilities to the four possible legal outcomes as a means of assessing overall system performance. It incorporates a utility matrix in which correct conviction and correct acquittal are each assigned a utility of +1; false acquittal is assigned a utility of −1; and false conviction is assigned a utility of −10. These values reflect the traditional notion that correct legal outcomes are more desirable that false outcomes and that false convictions are roughly ten times as bad as false acquittals. This node allows assessment of overall system performance using a common metric.

Fig. 5 also includes two nodes used to set key parameters of the system. These nodes allow the user of the network to test how the performance of the system varies as these parameters are varied. The “Prevalence” note sets the percentage of suspects being processed through the system who are guilty: high prevalence—90%; medium prevalence—50%; low prevalence—10%. In signal detection theory, what I am calling prevalence is often called the base rate. The “Decision Threshold” node sets the forensic examiner’s thresholds for identification and exclusion. The identification threshold can vary from 1.75 to 3.5 SD above the mean of the nonmatching distribution shown in Fig. 1; while the exclusion threshold can vary from 0 to 1.75 SD.

For the illustrations that follow, I will assume that the eyewitness has a correct ID rate of 0.80 and a false ID rate of 0.30. In other words, the eyewitness correctly identifies 80% of guilty suspects but falsely identifies 30% of innocent suspects. I will assume that the probability the forensic expert will identify, exclude or report an inconclusive when evaluating guilty and innocent suspects are those reflected in the signal detection model shown in Fig. 1 but with the assumption that the decision thresholds may vary.

## Forensic Decision Thresholds and the Risk of False Conviction

The model allows us to determine how the decision thresholds set by forensic examiners affect rates of true and false convictions in our hypothetical legal system. Table 1 shows how rates of true and false convictions vary for different decision thresholds. The ratio of true and false conviction rates constitutes a LR that describes the probative value of a conviction for distinguishing guilty and innocent suspects. In other words, in this legal system, how much more likely is a conviction for a guilty person than an innocent person?

**Table 1.** How true and false conviction rates vary for differing forensic decision thresholds

Threshold (Id/exclusion)	True conviction rate (% of guilty convicted)	False conviction rate (% of innocent convicted)	Ratio
3.50/1.75	53.2	0.61	87
3.25/1.50	58.0	1.01	57
3.00/1.25	62.4	1.61	39
2.75/1.00	66.0	2.43	27
2.50/0.75	69.6	3.48	20
2.25/0.50	72.0	4.80	15
2.00/0.25	74.4	6.30	11
1.75/0.00	76.4	8.10	9

Note: The identification threshold varies from 3.5 to 1.75 SD above the mean of the nonmatching distribution, while the corresponding exclusion threshold varies from 1.75 to 0 SD above that mean.

These findings highlight the importance of forensic decision thresholds. As the thresholds drop, the rate of true convictions increases but the rate of false convictions climbs much faster; hence, the ability of the system to distinguish guilty from innocent suspects decreases. These findings highlight the potential harm that can arise from contextual bias. If exposure to task-irrelevant information causes examiners to lower their thresholds, it increases the risk to innocent suspects and undermines the ability of the system to distinguish them from the guilty.

On the other hand, a high threshold also reduces the chances of correctly identifying a guilty person. Although the legal system generally considers it worse to convict the innocent than acquit the guilty, situations might conceivably arise in which the number of guilty persons acquitted is so high, relative to the number of innocents that are falsely convicted, that lowering the threshold for a forensic identification will increase rather than decrease the overall utility of legal outcomes. The Bayesian network model described here shows how that can happen when a high proportion of people being processed through the legal system are guilty.

It is worth noting that the proportion of people being processed through the system who are guilty has no effect on the probability that any given guilty person will be convicted, nor on the probability that any given innocent person will be convicted. The proportion of people who are guilty does, however, have a dramatic effect on the overall numbers of true and false convictions and true and false acquittals, as shown in Table 2. Table 2 shows how shifts in forensic examiners’ decision thresholds affect the percentage of verdicts falling in each of the four categories and the utility associated with each of those distributions.

**Table 2.** How the prevalence of guilt and the examiner’s decision threshold affect the percentage of verdicts falling in each of four categories and the utility of those outcomes

Threshold (ID/exclusion)	Correct convictions	Correct acquittals	False convictions	False acquittals	Utility
	10% Prevalence of guilt				
3.5/1.75	5.32	89.45	0.55	4.68	85
3.25/1.50	5.8	89.09	0.91	4.2	82
3.00/1.25	6.24	88.55	1.45	3.76	77
2.75/1.00	6.6	87.81	2.19	3.4	69
2.50/0.75	6.96	86.87	3.13	3.04	59
2.25/0.50	7.2	85.68	4.32	2.8	47
2.00/0.25	7.44	84.33	5.67	2.56	33
1.75/0.00	7.64	82.71	7.29	2.36	15
	50% Prevalence of guilt				
3.5/1.75	26.6	49.7	0.3	23.4	50
3.25/1.50	29	49.5	0.5	21	52
3.00/1.25	31.2	49.2	0.8	18.8	54
2.75/1.00	33	48.79	1.22	17	53
2.50/0.75	34.8	48.26	1.74	15.2	50
2.25/0.50	36	47.6	2.4	14	46
2.00/0.25	37.2	46.85	3.15	12.8	40
1.75/0.00	38.2	45.95	4.05	11.8	32
	90% Prevalence of guilt				
3.5/1.75	47.88	9.94	0.06	42.12	15
3.25/1.50	52.2	9	0.1	37.8	23
3.00/1.25	56.16	9.84	0.16	33.84	31
2.75/1.00	59.4	9.76	0.24	30.6	36
2.50/0.75	62.64	9.65	0.35	27.36	41
2.25/0.50	64.8	9.52	0.48	25.2	44
2.00/0.25	66.96	9.37	0.63	23.04	47
1.75/0.00	68.76	9.19	0.81	21.24	49

The first point to notice is that the examiner’s decision threshold affects the overall rates of conviction and acquittal. As the threshold decreases, the number of convictions increases and the number of acquittal declines, and this is true for both correct convictions and false convictions, and for both correct and false acquittals, regardless of the prevalence of guilt.

The second point to notice is that the prevalence of guilt has a powerful effect on the proportion of verdicts that are convictions and acquittals. As one would expect, acquittals greatly outnumber convictions where the prevalence of guilt is 10% and vice versa where the prevalence of guilt is 90%. Acquittals also outnumber convictions when the prevalence of guilt is 50%, although the difference is less striking.

The key finding, however, is the way in which variations in thresholds and prevalence affect the balance among the four outcomes, and in turn how that affects the measure of utility. When the prevalence of guilt is low (10%), the forensic examiner can maximize the utility of legal outcomes by adopting a high decision threshold. The utility score is highest (85) when the identification threshold is 3.5 SD and the exclusion threshold is 1.75 SD As these thresholds drop, the utility value also drops. By contrast, when the prevalence of guilt is high (90%), the maximum utility score (49) is achieved when the thresholds are much lower (1.75 SD for identification and 0.0 SD for exclusion). When the prevalence of guilt is intermediate (50%), utility is maximized (54) with thresholds of 3.00 SD for identification and 1.25 SD for exclusion. Interestingly, these thresholds are very close to those applied by examiners in the Ulery black-box study (12).

These findings make sense intuitively if one considers how the ratio between guilty and innocent suspects in the system affects the overall numbers of people falling in each of the verdict categories. When the prevalence of guilt is low, the number of innocent suspects at risk of being falsely convicted is much larger than the number of guilty suspects at risk of being falsely acquitted. It is therefore particularly important to minimize the risk of false conviction relative to the risk of false acquittal, and that requires high thresholds. By contrast, when the prevalence of guilt is high, there are many more guilty suspects at risk of false acquittal than innocent suspects at risk of false conviction. If examiners lower their identification thresholds in that situation, then the utility gain from increasing the chances of convicting the guilty people may exceed the utility loss from the increasing the risk of convicting innocents.

The gain in utility in the latter situation occurs, although the probative value of a conviction for distinguishing guilty from innocent drops dramatically as the identification threshold is reduced, as shown in Table 1. Innocent suspects face far greater risk when the thresholds are low. From the standpoint of utility maximization, however, it may be worth sacrificing the interests of a few innocents if doing so allows conviction of enough of the guilty.

## Discussion

Legal scholars have traditionally invoked notions of utility when discussing thresholds for conviction. The aphorism attributed to Blackstone that it is better ten guilty men go free than one innocent man be convicted is an explicit statement about the relative utility of alternative errors. Consequently, it seems natural to assume that a utilitarian calculus should guide decisions about setting thresholds for forensic identifications and exclusions. The findings of this article provide insights regarding the variables that affect such an analysis. Most importantly, this analysis highlights the importance of considering the prevalence of guilt in the population being evaluated. The optimal thresholds from a utilitarian perspective will vary depending on the relative numbers of guilty and innocent people in the population of potential suspects.

This raises the question, however, of whether utility maximization should be the sole consideration in setting decision thresholds. Legal scholar E.K. Cheng contends that the goal of the criminal justice system is not solely to maximize utility but also to keep the probability of a false conviction acceptably low—that is, to convict as many guilty people as possible without creating an undue risk to innocent people who are swept up in the system (26). According to Cheng, the legal systems of Western democracies have long recognized the importance of protecting the innocent from being mistakenly convicted and have traditionally accepted some loss of utility to achieve that goal. Cheng’s analysis suggests that the primary focus, when setting decision thresholds, should be minimizing the risk of false convictions. Under that approach, the prevalence of guilt would be a secondary consideration (if considered at all) because it is problematic to put an innocent person at risk, even if doing so greatly increases the ability of the system to convict the guilty.

Cheng makes a persuasive case that Western legal traditions favor minimizing risk to the innocent, but there is some evidence that the general public considers Blackstone’s 10:1 ratio too large (12, 27). While it is not clear how thoughtful such responses are, people say (when asked) that they think false acquittals are almost as bad as false convictions.

Because forensic scientists’ decision thresholds have such important implications for the legal system, forensic scientists should probably make decisions about threshold setting in conjunction with legal professionals. The optimal balance between false convictions and false acquittals is not a question that can be answered by science. It requires consideration of legal principles and social values. This article was written in the hope that it will bring the importance of forensic scientists’ decision thresholds to the attention of both the forensic science and legal communities and initiate a broader discussion of the issue.

Forensic scientists sometimes say that their goal is to be neutral regarding legal and policy issues, leaving such questions to the courts. The analysis presented here suggests, however, that there is no neutral position when it comes to setting decision thresholds. The threshold chosen, regardless of what it is, will affect the balance of risks in ways that have important implications for the legal system. Legal professionals should therefore be involved in making decisions about how thresholds are set.

The analysis reported here also casts light on the problem of contextual bias by showing how task-irrelevant contextual information may cause decision thresholds to shift and how such a shift would affect the accuracy of both the forensic examiner and the legal system. The key finding is that a downward shift in decision thresholds, which would be expected if examiners are exposed to incriminating contextual information, always degrades the probative value of a forensic identification by reducing the ratio of true identifications to false identifications. Lowering the decision threshold decreases the probative value of a forensic identification and decreases the ability of the legal system to distinguish the guilty from the innocent. That is why a forensic examiner who lowers decision thresholds to improve the chances of making the “right call” may paradoxically undermine the ability of the legal system to distinguish guilty from innocent defendants.

The utilitarian analysis presented here suggests that forensic examiners might, nevertheless, be able to improve system utility by varying their decision thresholds. If examiners could reliably apply higher thresholds when evaluating suspects who are less likely to be guilty, and lower thresholds when evaluating suspects who are more likely to be guilty, then they might be able to improve the overall utility of the legal system. Intuitions about this possibility may well underlie claims like those of Curley et al. (5) that taking account of task-irrelevant information can increase accuracy. This approach has a host of problems, however.

First, forensic examiners are not well positioned to evaluate the strength of other evidence against a suspect. They tend to rely entirely on information provided by police and prosecutors, which may be incomplete or slanted in favor of preferred interpretations. If examiners cannot evaluate the strength of the case against suspects accurately, it will be difficult for them to achieve any gains in utility from shifting thresholds according to perceived case strength. Second, as noted by the National Commission on Forensic Science (4), forensic scientists have no business engaging in such an analysis. Their job is to draw conclusions from physical evidence, not to evaluate the strength of the overall case. Third, as discussed above, lowering decision thresholds based on utility would place innocent suspects at greater risk of false incrimination, which arguably violates traditional values of the justice system. If forensic scientists decide to engage in case-specific threshold shifting, despite these problems, they should, at a minimum, disclose doing so in order that those who rely on their reports will understand the examiner’s decision threshold and its implications for the risk of error.

While this exercise in modeling illustrates the potential significance of examiners’ decision thresholds, it is important to acknowledge that, at present, we have limited information about how forensic examiners set their decision thresholds in actual casework. It seems likely that decision thresholds vary over time, both within and between examiners, even if other conditions remain the same. If the circumstances in which the judgment is being made also change (from study to casework and from one type of case to another) even greater variation may occur. One study found that the same examiners examining the same prints on two different occasions reach different conclusions about 10% of the time (28). There is also marked variation from study to study, and across disciplines, in the percentage of comparisons that pattern-matching examiners deem to be inconclusive (29). Hence, as noted earlier, it is possible, perhaps even likely, that forensic examiners’ decision thresholds in casework will differ from their thresholds when participating in black-box studies. That means that error rates observed in black-box studies may not provide accurate estimates of error rates in practice.

Concern about this issue led the American Association for the Advancement of Science (AAAS) to recommend, in its report on latent fingerprint analysis (3), that forensic laboratories “introduce known-source research samples into the routine flow of casework, so that examiners do not know their performance is being studied.” (p. 10). The AAAS noted that “[p]erceptions of the consequences of possible errors may well vary from setting to setting and from case to case in ways that greatly affect willingness to reach a definitive conclusion, and hence the probability of a false identification or false exclusion.” Citing the same concerns, the National Commission on Forensic Science (30) also called for “test blind” research in forensic laboratories and called upon government agencies to facilitate this kind of research. Feedback from such test blind studies could provide a more realistic assessment of examiners’ performance and would help examiners better understand their own accuracy and the risks of error associated with differing decision thresholds. The analysis reported here adds weight to those concerns by illustrating just how important the examiners’ decision threshold can be, and by showing that this issue has implications not just for forensic laboratories, but more broadly for the accuracy of the legal system.

In the absence of such research, it is problematic to draw inferences about error rates in forensic practice from current black-box studies. Commentators frequently claim that the error rates observed in black-box studies, such as the Ulery et al. study (13), represent the error rates for a forensic discipline. This claim has been questioned on grounds that the black-box studies do not mimic the conditions of casework (3, 10, 17), but the analysis reported here provides another reason to doubt this claim: It rests on the assumption that the decision thresholds applied by participants in the black-box study are the same as the decision thresholds applied in forensic practice. As noted earlier, there is substantial reason to doubt this assumption. Examiners may in fact apply different decision thresholds in different circumstances without even being aware of doing so, particularly if they are exposed to potentially biasing task-irrelevant information. When one considers this possibility, in connection with this article’s demonstration that even small changes in decision thresholds can have a dramatic effect on error rates, it becomes clear that error rates in forensic practice may well differ substantially from the error rates observed in black-box studies.

Should forensic scientists consider dispensing with decision thresholds? Rather than following the American approach of evaluating the similarity of the items under comparison and then deciding whether they are sufficiently similar to report an identification (or other categorical conclusion), some commentators have suggested examiners could follow the European approach, which eschews categorical conclusions and requires examiners instead to make a statement about the strength of the evidence for supporting (or contradicting) the hypothesis that the items have a common source. For example, examiners might present a number representing their subjective judgment of the relative likelihood of the observed similarities (and dissimilarities) under alternative hypotheses about the source of the items (i.e., same-source or different source). Alternatively, or additionally, examiners might make some verbal statement about the strength of the evidence that is based on their subjective evaluation of the relative probability of the observed results under the alternative hypotheses. For example, rather than saying that she has identified the latent print as being from the same finger as a reference print, the examiner might say that she believes the observed correspondence in the print patterns is one million times more probable if the prints were made by the same finger than if they were made by different fingers. Alternatively, or additionally, the examiner might say that the comparison provides extremely strong evidence that the prints have a common source.

A common objection to the European approach is that forensic examiners at present have an insufficient scientific basis for assessing the probability of their observations under the same-source or different-source hypotheses. The LRs they present in reports and testimony (and the verbal statements based thereon) may be sincere statements about their subjective beliefs, but the method has not been adequately validated: We do not know whether and to what extent examiners’ assessments of probability are trustworthy (31).

While this is a fair criticism, it is important to recognize that the American approach suffers from the same deficiency in validation. If there is an insufficient basis for assessing the probability of the observed evidence under the relevant hypotheses, that problem affects the trustworthiness of the evidence regardless of how the examiner reports findings. The choice is between a statement of uncertain merit about the strength of the evidence or a statement of uncertain accuracy about the examiner’s categorical conclusion.

A possible advantage of the European approach is that it makes the probabilistic basis of the evidence, and its underlying uncertainty, more transparent; while the American approach tends to hide this problem (32, 33). The major disadvantage of the European approach is that recipients may find statements about LRs or strength of support more difficult to understand than a simple categorical conclusion (2, 3, 33).

A key issue is whether examiners who take the European approach will be less susceptible to contextual bias. This claim is plausible because the European approach eliminates the need for examiners to set a decision threshold and thus eliminates the possibility that bias may arise through a threshold shift. It is possible, however, that exposure to contextual information might influence examiners’ estimates of the conditional probabilities of the observed evidence under the hypotheses. Thus, an examiner who hears that an eyewitness has identified a suspect may tend to give higher estimates of the LR and describe the evidence as providing stronger support for the same source hypotheses. Additional research to explore this possibility would be worthwhile. In the meantime, laboratories wishing to minimize the potential for contextual bias should consider adopting blinding procedures, regardless of the reporting format.

Finally, it is important to acknowledge the limitations of the analysis presented here. This analysis relied on simple conceptual models: a signal detection model representing the decision-making process of forensic examiners and a Bayesian network model showing how the thresholds applied in the forensic analysis might affect rates of conviction and acquittal in a simplified hypothetical legal system. My goal was to illustrate conceptually how decision thresholds affect the probative value of forensic evidence and how shifts in threshold might affect rates and ratios of true and false convictions in a simplified legal system. I do not claim that the rates of error shown in these models, particularly rates of true and false convictions, correspond to actual error rates in the real legal system, as outcomes in the actual legal system are obviously determined by many additional factors that may increase or decrease the risk of error. This article was written to illustrate principles that will apply in any legal system, not to provide an accurate representation of error rates in any particular jurisdiction.

## Data Availability

There are no data underlying this work.
